# Isolation and characterization of βA3-crystallin associated proteinase from α-crystallin fraction of human lenses

**Published:** 2008-10-20

**Authors:** O. P. Srivastava, K. Srivastava, J. M. Chaves

**Affiliations:** Department of Vision Sciences, University of Alabama at Birmingham, Birmingham, AL

## Abstract

**Purpose:**

The purpose was to characterize the properties of a proteinase activity associated with βA3-crystallin, which was isolated from the α-crystallin fraction of human lenses.

**Methods:**

An inactive, Arg-bond hydrolyzing proteinase in the α-crystallin fraction, which was isolated from the water soluble (WS) protein fraction of 60- to 70-year-old human lenses, was activated by sodium deoxycholate treatment. The activated enzyme was purified by a three-step procedure that included a size-exclusion Agarose A1.5 m chromatography, non-denaturing preparative gel-electrophoresis, and size-exclusion HPLC. The purified proteinase was characterized for the proteinase type, proteolysis of bovine recombinant γB-, γC-, and γD-crystallins, and its presence in three different protein fractions of human lenses (i.e., α-crystallin, β_H_-crystallin, and membrane fractions).

**Results:**

An inactive, Arg-bond hydrolyzing proteinase present in the α-crystallin fraction showed activity on treatment with detergents such as sodium deoxycholate, Triton X-100, octyl β-D-glucopyranoside, and CHAPS (3-[(3-cholamido propyl) dimethylammonio]-1-propanesulfonate). The sodium deoxycholate-activated enzyme was released from the α-crystallin fraction since it eluted at a lower molecular weight species than α-crystallin during size-exclusion Agarose A1.5 m chromatography. Following a three-step purification procedure, the enzyme showed three species between 22 kDa and 25 kDa during sodium dodecyl sulfate-polyacrylamide gel electrophoresis (SDS–PAGE) analysis. The three protein bands were identified as βA3-, βB1-, and βB2-crystallin by the matrix-assisted laser desorption/ionization-time of flight (MALDI-TOF) and tandem mass spectrometric (ES-MS/MS) methods. Inhibitor studies revealed that the enzyme was a serine-type proteinase. Among the recombinant βA3-, βB1-, or βB2-crystallins, only the βA3-crystallin exhibited the proteinase activity following detergent treatment and size-exclusion chromatography. The proteinase also exhibited proteolysis of γC- and γD- crystallins, and the cleavage of γD-crystallin at M_1_-G_2_, Q_54_-Y_55_, M_70_-G_71_, and Q_103_-M_104_ bonds. Further, the enzyme was also present in three fractions of human lenses (α-crystallin, β_H_-crystallin, and membrane fractions).

**Conclusions:**

A serine-type βA3-crystallin proteinase existed in an inactive state in the α-crystallin fraction and was activated by detergents. The enzyme proteolyzed αA-, αB-, γC-, and γD-crystallins and was present in three fractions (α-crystallin, β_H_-crystallin, and membrane-fractions) of 60 to 70-year-old human lenses.

## Introduction

The vertebrate lens contains several endopeptidases such as a neutral proteinase (a multicatalytic proteasome) [1,2], a 25 kDa proteinase [3], calpain I and calpain II [4], an Lp82 calpain [5], a membrane-associated proteinase [6,7], a Ca^+2^-dependent protease [8], and caspase-3 and caspase-6 [9-11]. Additionally, the existence of ubiquitin-proteasome activity has been described in the lens [12]. In spite of the existence of so many proteinases, the lens exhibits a low protein turnover rate [13], which is further suggested by the limited degradation of α-, β-, and γ-crystallins in mammalian lenses. However, truncation of the crystallins increases with aging and cataractogenesis in human lenses for reasons yet unknown [14-19].

Our previous studies have shown that the human lens 25 kDa proteinase [3] and a membrane proteinase [6,7] hydrolyzed the Arg-bond-containing substrates. More recently, we presented evidence that the Arg-bond hydrolyzing proteinase activity was associated with βA3/A1-crystallin [20]. The properties of the enzyme were apparently similar to a previously studied 25 kDa proteinase [3] and a membrane proteinase [6,7], both of which needed activation and were a serine-type. The results suggested that the βA3/A1-crystallin proteinase might be present in water soluble fractions and associated with the membranes in human lenses.

Our previous results [3,6,7,20] have also shown that the Arg-bond hydrolyzing lens proteinase existed in an inactive state and was activated by either metal ions [3] or by a detergent such as sodium deoxycholate [20]. Although the proteinase existed in an inactive state possibly due to its inhibition by α-crystallin as suggested in previous studies [21-27] and in the reports that show inhibition of trypsin [24] and elastase [26] by α-crystallin, the presence of the enzyme in an inactive state in the α-crystallin fraction has not been investigated.

With the exception of our report of βA3-crystallin as the proteinase [20] and the observation that recombinant βA3-crystallin readily degrade to 20–25 kDa major species during purification and on storage, no other report has suggested such an association between enzyme activity and the crystallin. Nevertheless, suggestive evidence exists in one report [28], which showed that βA3/A1-crystallin, upon ultraviolet (UV) irradiation, exhibited degradation and multimer formation in species higher than 27 kDa. Although the report attributed the disappearance of the crystallin to increasing doses of irradiation, the observed pattern was similar to what we have observed during sodium deoxycholate-induced activation of proteinase activity in βA3-crystallin. Presently, the molecular events that lead to the activation of proteinase activity in the crystallin is not fully understood, except that the truncation in the NH_2_-terminal extension and altered conformation of the crystallin are required. The age-related NH_2_-terminal truncation in βA3/A1-crystallin has been demonstrated in fetal and adult bovine lenses with the absence of NH_2_-terminal 11 and 22 amino acids [29]. Similarly, NH_2_-terminal truncations of βB1- and βA3/A1-crystallins in three-year-old human lenses was also reported [30], and we showed that age-related progressive NH_2_-terminal truncations in human βA3-crystallin produce 4-18 kDa species with a major cleavage at the E_39_-N_40_ bond [15]. The potential effects of these in vivo NH_2_-terminal truncations on crystallin structure and proteinase function have not been studied.

Based on our previous results as described above, we hypothesized that because α-crystallin acts as an inhibitor of βA3-crystallin proteinase, a proteinase/α-crystallin complex might exist in the α-crystallin fraction of human lenses. To test this hypothesis, we used the α-crystallin fraction to activate and purify the proteinase and examined whether the enzyme activity was associated with βA3-crystallin. In addition, we have also examined whether this enzyme exists in the α-crystallin, β_H_-crystallin, and membrane fractions of human lenses.

## Methods

### Materials

Normal human lenses with no apparent opacity were obtained from Dr. Robert Church (Emory University, Atlanta, GA). The retrieved lenses were stored at −20 °C in Medium 199 (without phenol red) until used. The prestained and unstained molecular weight markers were from GE Biosciences (Piscataway, NJ). Unless indicated otherwise, all the other chemicals used in this study were purchased from either Sigma (St. Louis, MO) or Fisher (Atlanta, GA). The recombinant βA3/A1-crystallin was expressed in *Escherichia coli* and purified in our laboratory as previously reported [31]. The recombinant βB1- and βB2-crystallins were a gift from Dr. Kirsten Lampi (Oregon Health Sciences University, Portland, OR).

### Isolation of human lens α-crystallin fraction

The water soluble (WS) protein fraction from 20 pooled lenses of 60 to 70-year-old donors was prepared as previously described [17,32]. Briefly, each lens was thawed on ice, decapsulated, suspended (2 ml/lens) in buffer A (50 mM Tris-HCl, pH 7.9, containing 1 mM dithiothreitol [DTT], 1 mM iodoacetamide, 1 mM phenylmethylsulfonyl fluoride), and homogenized using a tissue grinder. The lens homogenate was centrifuged at 25,000x g for 15 min. The supernatant was recovered and the pellet was homogenized and centrifuged twice as described above. The supernatants recovered after each centrifugation were pooled and designated as the WS protein fraction, and the pellet was designated as the water insoluble (WI) protein fraction. The WS protein fraction was subjected to size-exclusion chromatography using an Agarose A5 m column (2.5×40 cm) to recover the high molecular weight (HMW) protein fraction as well as α-, β_H_-, β_L_-, and γ-crystallin fractions. The individual column fractions were analyzed by sodium dodecyl sulfate-polyacrylamide gel electrophoresis (SDS–PAGE) to identify the presence of specific crystallins, and each of the fractions were individually pooled, dialyzed against a desired buffer, and stored at −20 °C before use.

### Proteinase activity in α-crystallin fraction

The Arg-bond hydrolyzing proteinase activity was measured by the hydrolysis of either N-benzoyl DL-Arg p-nitroanilide (BAPNA) or CBZ-L-Arg 7-amino 4-methyl coumarin as a substrate (see below the method for enzyme assay) in the α-crystallin and in other desired protein preparations. The proteinase activity in the α-crystallin fraction was determined by the following three methods: (1) The α-crystallin fraction was treated with 2% sodium deoxycholate (w/v), and the activity was determined in the detergent-treated and untreated fractions following size-exclusion Agarose A 1.5 m chromatography; (2) the α-crystallin was treated with individual detergents (sodium deoxycholate, sodium dodecyl sulfate [anionic], Triton X-100, octyl β-D-glucopyranoside [non-ionic], or 3-[(3-Cholamidopropyl) dimethylammonio]-1-propanesulfonate [CHAPS; a zwitterion]), and the proteinase activity was determined; or (3) the sodium deoxycholate-treated and untreated α-crystallin fractions were incubated at 37 °C for 10 h and then subjected to two-dimensional gel electrophoresis as described previously [15]. The polypeptide spots that were present only in the detergent-treated α-crystallin preparation were identified, excised, and analyzed by the matrix-assisted laser desorption/ionization-time of flight (MALDI-TOF) mass spectrometric method.

### Purification of the Arg-bond hydrolyzing proteinase from the α-crystallin fraction

An Arg-bond hydrolyzing proteinase was purified by a three-step procedure from the α-crystallin fraction of normal lenses of 60 to 70-year-old donors. All the purification steps were performed at 5 °C unless indicated otherwise. In the first step, the α-crystallin fraction was prepared as described above from the WS protein fraction by a size-exclusion Agarose A 1.5 m chromatography, treated with sodium deoxycholate (final concentration of 2% [w/v]), and chromatographed through an Agarose A1.5 m column (5×80 cm). The column equilibration and sample elution were performed with buffer A (see above for the composition). In the second step, the proteinase-containing fractions from the Agarose column were pooled, concentrated by lyophilization, and dialyzed against 10X excess of buffer A. The preparation was then subjected to preparative non-denaturing gel (10% polyacrylamide gel) electrophoresis using a Bio-Rad Prep Cell  (model 491; Hercules, CA). The fractions were recovered as proteins that were eluted from the preparative gel and analyzed for enzyme activity with BAPNA as a substrate and for protein species by SDS–PAGE [33]. The enzyme-containing fractions were pooled, concentrated by lyophilization, and dialyzed against 0.05 M phosphate buffer, pH 7.5. In the third step, the proteinase-containing fractions were fractionated by HPLC using a size-exclusion TSK G3000 PW_XL_ column (TosoHaas, Montgomeryville, PA). During HPLC, the column equilibration and sample elution were performed with 0.05 M phosphate buffer, pH 7.5. The proteinase-containing fractions were pooled, dialyzed against a 2,000 volume excess of phosphate buffer at 5 °C for 48 h, and stored at −20 °C.

### Identification of protein species in the purified proteinase preparation

The purified proteinase preparation was examined by SDS–PAGE and the identity of the protein bands were established by MALDI-TOF and the tandem mass spectrometric (ES-MS/MS) methods. MALDI-TOF MS analysis and ES-MS/MS sequencing (Micromass Q-TOF 2; Waters-Micromass, Manchester, UK) were performed at the Comprehensive Cancer Center Mass Spectrometry Shared Facility of the University of Alabama at Birmingham. For mass spectrometric analysis, the individual protein spots/bands were excised from a SDS–PAGE gel using pipette microtips. The polyacrylamide pieces containing individual bands were destained with three consecutive washes with a 50:50 mixture of 25 mM ammonium bicarbonate and acetonitrile for 30 min. Next, the samples were washed for 10 min with 25 mM ammonium bicarbonate before digestion with trypsin (12 ng/μl; sequencing grade from from Roche, Applied Sciences, Indianapolis, IN) for 16 h at 37 °C. Peptide solutions were then extracted using 100 μl of a 50:50 mixture of 5% formic acid and acetonitrile for 30 min. Supernatants were collected and evaporated to dryness in a Savant SpeedVac (Marietta, OH). Samples were resuspended in 10 μl of 0.1% formic acid. C-18 ZipTips (Millipore, Billerica, MA) were used to desalt peptide mixtures before applying samples to the MALDI-TOF 96 well spot, gold-coated target plates. Bound peptides were eluted from the ZipTips with 10 μl of 80% acetonitrile containing 0.1% formic acid. They were mixed (1/10 dilution) with a saturated solution of α-cyano-4-hydroxycinnamic acid (CHCA) matrix in 50% aqueous acetonitrile. Samples were allowed to dry before performing MALDI-TOF MS using a Voyager DE-Pro (ABI, Foster City, CA) with a nitrogen laser (337 nm) operating in positive mode. Spectra were the sum of 100 laser shots – they were then de-isotoped and analyzed using Voyager Explorer Software (Applied Biosystems, Forest City, CA), and peptide masses were submitted to the MASCOT search engine for protein identification. The potential identity of the proteins was determined by using the NCBInr database (National Center for Biotechnology Information, Bethesda, MD). Tandem mass spectral analyses were performed with the Q-TOF 2 mass spectrometer using electrospray ionization to verify the identity of the proteins. The tryptic peptides used for the MALDI-TOF MS analysis were then analyzed by LC-MS/MS. Liquid chromatography was performed using a LC Packings Ultimate LC-Switchos microcolumn switching unit and Famos autosampler (LC Packings, San Francisco, CA). The samples were concentrated on a 300 μm i.d. C_8_ reverse-phase precolumn at a flow rate of 10 μl/min with 0.1% formic acid and then flushed onto a 10 cm×75 μm i.d. C_8_ reverse-phase column at 200 μl/min with a gradient of 5%–100% acetonitrile in 0.1% formic acid for 30 min. The nanoelectrospray ionization interface was used to transfer the LC eluent into the mass spectrometer. The Q-TOF was operated in the automatic switching mode where multiple-charged ions were subjected to MS/MS if their intensities rose above six counts. Protein identification was performed by either ProteinLynx Global Server software (Waters, Milford, MA) or by manual interpretation.

### Additional studies

#### Nature of the α-crystallin-associated proteinase

A variety of inhibitors were used to determine the nature of the proteinase. These included phenylmethylsulfonyl fluoride (PMSF; 1 mM), diisopropyl fluorophosphate (DFP; 1 mM), and benzamidine (10 mM) as serine proteinase inhibitors; ethylenediamine tetraacetic acid (EDTA; 10 mM) and diethyldithiocarbamic acid (10 mM) as metalloproteinase inhibitors; and iodoacetamide (3 mM) and iodoacetic acid (3 mM) as cysteine proteinase inhibitors. Percent inhibition of the enzyme by an inhibitor was determined following incubation with BAPNA at 37 °C for 30 min.

#### Structural changes in βA3-crystallin following proteinase activation on sodium deoxycholate treatment

The purified recombinant His-tagged wild type (WT) βA3 (M_r_ ~32 kDa) was treated with 0.1% sodium deoxycholate and fractionated by size-exclusion HPLC using a TSK G3000 PW_XL_ column. The column fractions containing proteinase activity were analyzed by SDS–PAGE. The fractions representing the highest proteinase activity peak and a detergent-untreated βA3-crystallin preparation were analyzed by SDS–PAGE. The partial NH_2_-terminal sequence of the major bands in the two fractions was determined at the Iowa State University Protein Core Facility (Ames, IA). The detergent-untreated and detergent-treated βA3-crystallin preparations with proteinase activity were used for the determination of structural changes as previously described [31]. The analyses included surface hydrophobicity changes by ANS (8-anilino-1-naphthalenesulfate, a hydrophobic probe)-binding, Trp microenvironmental changes by intrinsic Trp fluorescence spectra recording, and secondary structural changes by circular dichroism (CD) spectral determination. The fluorescence spectra were recorded in corrected spectrum mode using a spectrofluorometer (Shimadzu Scientific Instruments, Columbia, MD) with excitation and emission band passes set at 5 nm and 3 nm, respectively. The binding of a hydrophobic probe, ANS, to βA3-crystallin preparations was determined by recording fluorescence spectra after excitation at 390 nm and emission between 400 and 600 nm. In these experiments, 15 μl of 0.8 mM ANS (dissolved in methanol) was added to a protein preparation (0.1 mg/ml, dissolved in 10 mM phosphate buffer, pH 7.4). The samples were incubated at 37 °C for 15 min before the fluorescence measurements. The intrinsic Trp fluorescence intensities of the βA3-crystallin preparations (0.1 mg/ml of each) were recorded with an excitation at 295 nm and emission between 300 and 400 nm. The secondary structural changes in the βA3-crystallin preparations were determined by recording their far-UV CD spectra at room temperature using an AVIV Spectropolarimeter (Model62DS; AVIV, Lakewood, NJ). The βA3-crystallin preparations of 0.1–0.2 mg/ml (dissolved in 50 mM Tris-HCl, pH 7.9) were used for recording the far-UV CD spectra. The path length was 0.1 cm during the far-UV CD spectra determination. The spectra reported are the average of five scans, corrected for buffer blank and smoothed. Secondary structures were estimated using the SELCON program [34].

#### Determination of the Arg-bond hydrolyzing proteinase activity in water soluble protein, α-crystallin, β_H_-crystallin, and membrane fractions

The WS protein fraction was isolated from six pooled lenses from 30 to 40-year-old donors and the α-, β_H_-, β_L_-, and γ-crystallin fractions were isolated by Agarose A1.5 m column chromatographic method as described above. Individual α-, β_H_-, β_L_-, and γ-crystallin species were collected following SDS–PAGE analysis of the column fractions. The WI protein fractions from these lenses were used to isolate the membrane fraction by a urea wash method as previously described [6].

To activate proteinase activity, the individual WS protein, α-, and β_H_-crystallin fractions were treated with sodium deoxycholate (2% w/v, final concentration) and fractionated by an Agarose A1.5 m column. The column fractions were analyzed for the Arg-bond hydrolyzing proteinase activity using BAPNA as a substrate. The membrane fraction was also extracted with 2% (w/v; final concentration) sodium deoxycholate for 20 h at 4 °C, centrifuged at 15,000x g for 15 min, and the supernatant was chromatographed as above to determine the enzyme activity. The enzyme preparations isolated from each of the four fractions (i.e., WS protein, α-crystallin, β_H_-crystallin, and membrane fractions) were further subjected to nondenaturing polyacrylamide (10%) gel electrophoresis using Bio-Rad Prep Cell (model 491), and the enzyme-containing fractions were collected, concentrated by lyophilization, dialyzed at 4 °C against 0.05 M phosphate buffer, pH 7.5, and stored frozen at −20 °C until use.

To determine whether a single proteinase exist in the above four fractions of human lenses, the following experiments were performed: (1) The elution of proteinase following Agarose A1.5 m chromatography of the above four fractions were compared to determine whether the enzyme had identical M_r_ and, therefore, coeluted in the identical fractions during chromatography; (2) a further confirmation of an identical M_r_ of the proteinase in the above four fraction was performed by a size-exclusion HPLC using a TSK G-4000 PW_XL_ column. The individual proteinase preparations that were recovered from the four fractions after Agarose A1.5 m chromatography were analyzed by HPLC, and the proteinase activity was determined. Again, the column fractions were analyzed as above for the enzyme activity; (3) to further determine the presence of a single proteinase, the individual enzyme preparations that were recovered following Agarose A1.5 m chromatography of the four fractions were combined and subjected to a preparative nondenaturing gel-electrophoresis with 10% polyacrylamide gel using a Bio-Rad Prep Cell (Model 491). The eluent from the gel was collected in different fractions and analyzed for the proteinase activity as above with BAPNA as a substrate; and (4) the purified proteinase preparations from the above four fractions were analyzed for their caseinolytic activity by the method as described [35]. The purpose of this experiment was to determine whether the enzyme recovered from the four fractions shows comigrating caseinolytic bands on a gel during nondenaturing gel electrophoresis.

#### Proteolysis of bovine recombinant γB-, γC-, and γD-crystallins by βA3-crystallin proteinase

Bovine recombinant γB-, γC-, and γD-crystallins (obtained from Dr. Mark Petrash, Washington University, St. Louis, MO) were incubated with the proteinase (isolated from the membrane fraction) for 48 h at 37 °C at the ratio of 1:40 (proteinase to crystallin). Aliquots withdrawn at 0 h and after 24 and 48 h of incubation were analyzed by SDS–PAGE. The proteolyzed species were transferred to a PVDF membrane [36], briefly stained with Coomassie blue, and individual proteolyzed species were analyzed for their partial NH_2_-terminal sequences at the Core Facility of the University of Alabama at Birmingham.

### Miscellaneous methods

The proteinase activity was assayed with two methods as previously described [3,6,7]. In the first method, benzoyl DL-Arg p-nitroanilide (BAPNA) was used as a substrate, and the assay was performed for 4 h or for 16-24 h at 37 °C. In the second fluorometric assay, a 3.0 ml reaction mixture contained 0.1 ml of 0.1 mM coumarin substrate solution (CBZ-L-Arg 7-amino 4-methyl coumarin, excitation at 380 nm and emission at 460 nm), 0.1 ml of enzyme preparation and 2.8 ml of 0.05 M Tris-HCl, pH 7.8. The reaction mixture was incubated at 37 °C for 5 min and enzyme activity determined using a Turner model 430 spectrofluorometer (Sequoia-Turner Corporation, Mountainview, CA), water-jacketed cuvette holder and Houston chart recorder (Houston Instrument, Austin, TX). With the coumarin substrate, one enzyme unit (e.u.) is defined as the production of 0.1 nanomole of 7-amido-4-methyl coumarin per min. The use of a specific substrate for the enzyme assay in the experiments has been identified.

The pI (isoelectric point) of the purified proteinase was determined using a Bio-Rad isoelectric focusing apparatus (Rotofor Preparative IEF Cell). Protein determination was performed using the Pierce protein determination kit (Pierce Chemical Co., Rockford, IL) using the manufacturer’s supplied method.

## Results

### An Arg-bond hydrolyzing proteinase exists in the α-crystallin fraction

**Figure 1:**
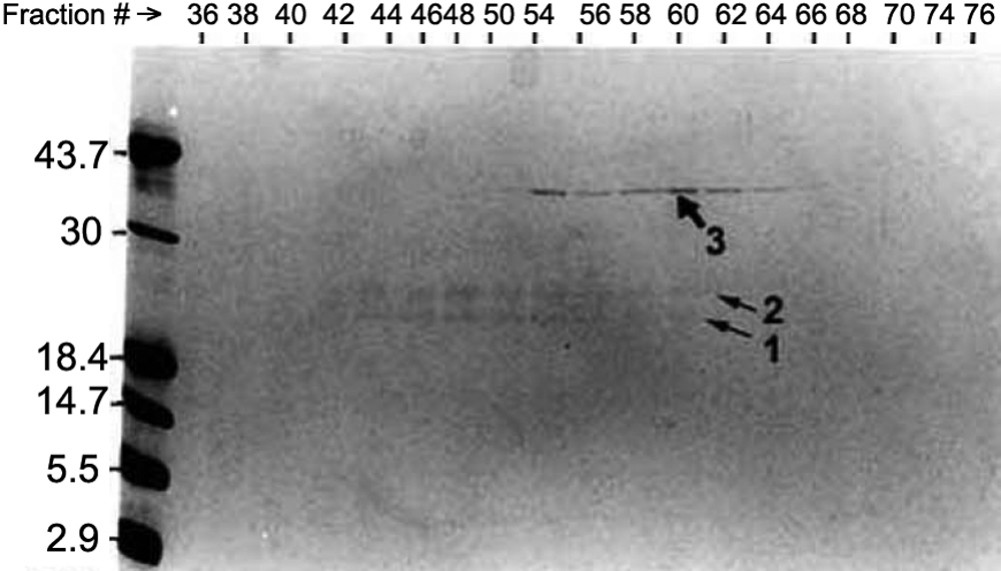
SDS–PAGE analysis of the column fractions that contained proteinase activity after Agarose A1.5 m chromatography of the sodium deoxycholate-treated α-crystallin fraction. Note that two protein bands (identified as bands 1 and 2) with M_r_ of 20–25 kDa and the third band (identified as band 3) of 38 kDa were recovered.

**Figure 2:**
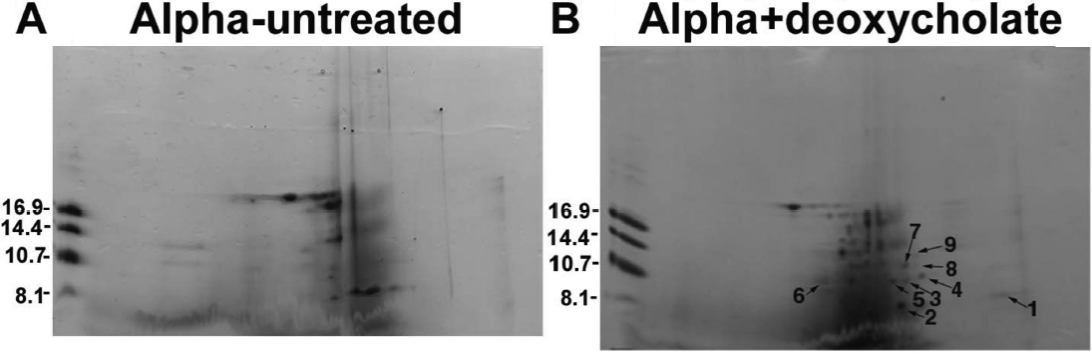
Proteolysis of αA- and αB-crystallins following incubation of the α-crystallin fraction with sodium deoxycholate at 37 °C for 10 h. Deoxycholate untreated α-crystallin fraction (**A**)  and deoxycholate (2% w/v)-treated α-crystallin fraction (**B**; each containing 190 μg protein at 37 °C for 10 h) were analyzed by two-dimensional gel electrophoresis, and those spots present in only the deoxycholate-treated preparations were identified and numbered as 1-9 as shown in (**B**). Each spot was analyzed by the MALDI-TOF mass spectrometric method to determine their identity.

The following three experiments suggested the presence of an Arg-bond hydrolyzing proteinase in the α-crystallin fraction of human lenses. In the first experiment, no proteinase activity was observed in the α-crystallin fraction of human lenses of 60 to 70-year-old donors until the preparation was treated with sodium deoxycholate and fractionated by a size-exclusion Agarose A1.5 m column. Following chromatography, the proteinase eluted later than α-crystallin, which suggests its dissociation and a lower molecular weight than the crystallin. On examination of the fractions that contained proteinase activity by SDS–PAGE, three protein bands, two of about 22–25 kDa and one of about 38 kDa, were observed (Figure 1). Similar results were obtained with the α-crystallin fraction isolated from lenses of 20-year-old donors, suggesting that the association of the enzyme with the crystallin occurred early during aging (results not shown). In the second experiment, the dissociation of the proteinase activity from the α-crystallin fraction following treatment with sodium deoxycholate (anionic, 0.1% final concentration), Triton X-100 (non-ionic, 0.1%), octyl β-D-glucopyranoside (non-ionic, 0.2%), and CHAPS (zwitterionic, 0.1%) was also observed. All other detergents except Triton X-100 were able to dissociate the enzyme at almost the same levels (1.0 enzyme units from 0.8 mg protein per ml from the α-crystallin fraction; Table 1). The third experiment showed that the activation of the enzyme also resulted in the proteolysis of α-crystallin. With the sodium deoxycholate treatment of α-crystallin and incubation under sterile conditions at 37 °C for 10 h, the α-crystallin preparations showed proteolysis, which was absent in the detergent-untreated preparation (Figure 2A,B). Upon MALDI-TOF mass spectrometric analysis, the proteolyzed fragments of both αA- and αB-crystallins were identified (i.e., spot no. 6 [M_r_ 10 kDa] was of αB-crystallin and spot no. 1-5 and 7-9 [M_r_ 6–14 kDa] were of αA-crystallin).

**Table 1:** Dissociation of proteinase activity from α-crystallin fraction.

**Detergents concentration**	**Type**	Proteinase activity (e.u./ml)*
Sodium deoxycholate (1%)	Anionic	1.04
Sodium dodecyl sulfate (0.1%)	Anionic	1.05
Triton X-100 (0.1%)	Non-ionic	0.5
Octyl-β-D-glucopyranoside (0.2%)	Non-ionic	0.8
CHAPS (0.2%)	Zwitterion	1.05

The proteinase activity was determined following detergent treatment of α-crystallin fraction. The asterisk indicates that proteinase activity is expressed as e.u./ml with BAPNA as a substrate. One e.u. is defined as the release of 1 nm/h of p-nitroanilide from BAPNA. Proteinase activity was determined by detergent treatment of the α-crystallin fraction followed by size-exclusion HPLC using a TSK G-3000 PW _XL_ column.

### Purification of an Arg-bond hydrolyzing proteinase from α-crystallin fraction

**Figure 3:**
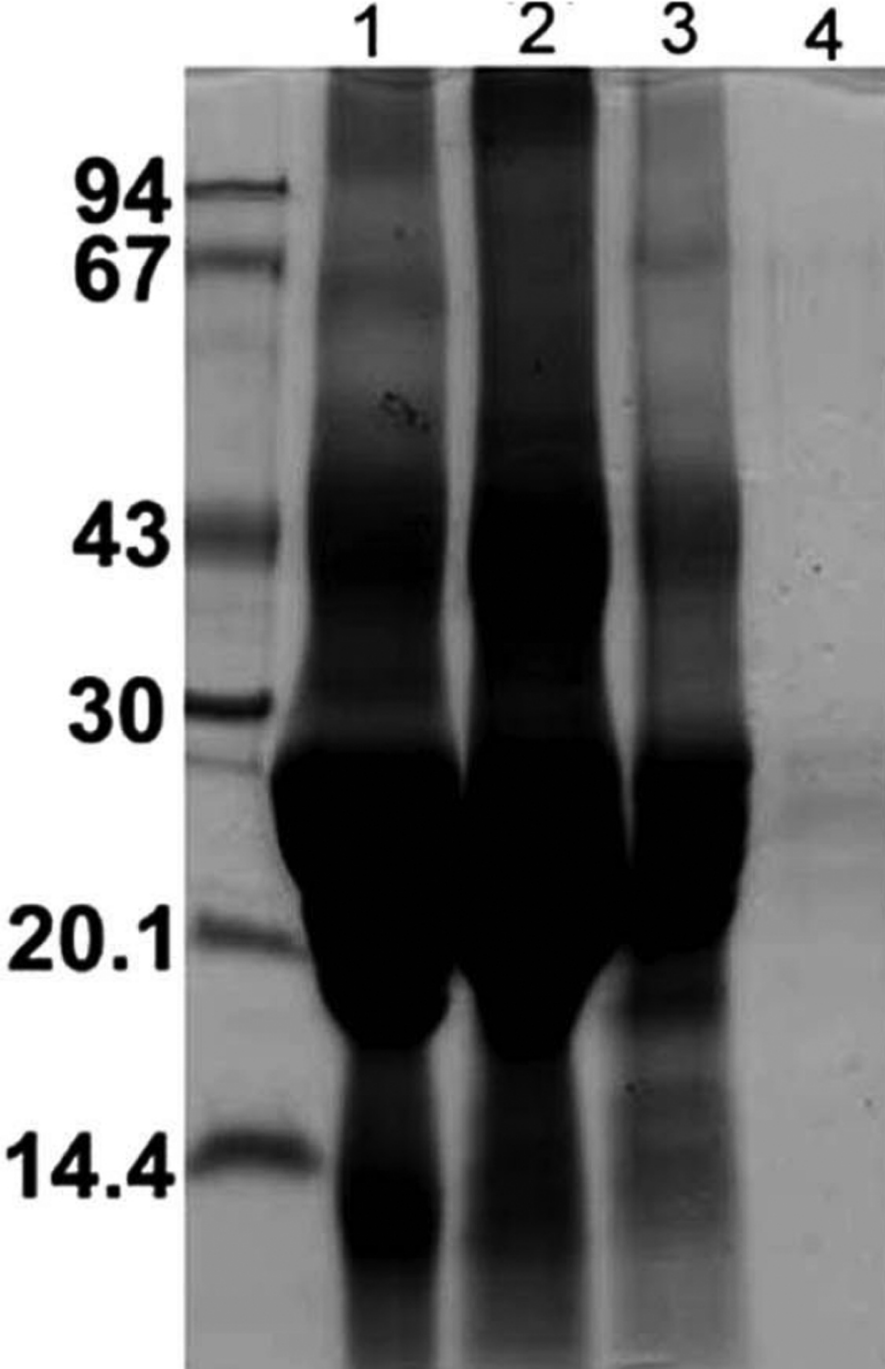
SDS–PAGE analysis of various fractions recovered during purification of α-crystallin-associated fractions. Lane 1: α-Crystallin fraction was recovered from a WS protein fraction of lenses that were 60- to 70-years-old. Lane 2: Concentrated α-crystallin fraction applied to the Agarose A1.5 m column. Lane 3: Proteinase fractions recovered following non-denaturing gel electrophoresis of the proteinase fraction released after Agarose A1.5 m gel chromatography. Lane 4: A proteinase-containing fraction recovered following size-exclusion HPLC using TSK G-4000 PW_XL_ column of the fraction recovered in lane 3.

The α-crystallin fraction, isolated from 60 to 70-year old human lenses, was used for the dissociation and purification of the enzyme by a three-step procedure (see Methods). Following sodium deoxycholate treatment and size-exclusion Agarose A1.5 m chromatography, the enzyme was released, and the proteinase-containing fractions showed a band of about 38 kDa and two bands of 22–25 kDa (Figure 1). The 38 kDa band was identified as keratin by MALDI-TOF mass spectrometric analysis. Upon further purification of the proteinase by the second step of nondenaturing gel-electrophoresis followed by the third step of size-exclusion HPLC, the 22–25 kDa species were progressively purified (Figure 3). Further, no additional species greater than 90 kDa were present in the purified fraction as determined by using 7% polyacrylamide gel during SDS–PAGE analysis (results not shown).

The specific activity and fold purification at various steps of enzyme purification are shown in Table 2. The specific activity of the proteinase after the three-step purification was 500 with a 1,094 fold increase in specific activity.

**Table 2:** Purification of α-crystalllin-associated proteinase from 60 to 70-year-old human lenses.

**Fractions**	**Protein (mg/ml)**	Enzyme (e.u./ml)*	Specific activity**
1. α-crystallin-sodium deoxycholate-treatment	3.5	1.6	0.457
2. Agarose A 1.5 m chromatography	0.44	14.8	33.6
3. Non-denaturing gel electrophoresis	0.08	2.4	30
4. Size-exclusion HPLC	0.004	2	500

Proteinase activity was determined following detergent treatment of the α-crystallin fraction and size-exclusion HPLC using a TSK G-3000 PW_XL_ column. An asterisk indicates that one enzyme unit (e.u.) is defined as the production of 0.1 nanomole of 7-amino 4-methyl coumarin per ml. The double asterisk denotes that specific activity is given in the measurement of e.u./mg of the protein.

### Identification of the protein species in the purified proteinase preparation

**Figure 4:**
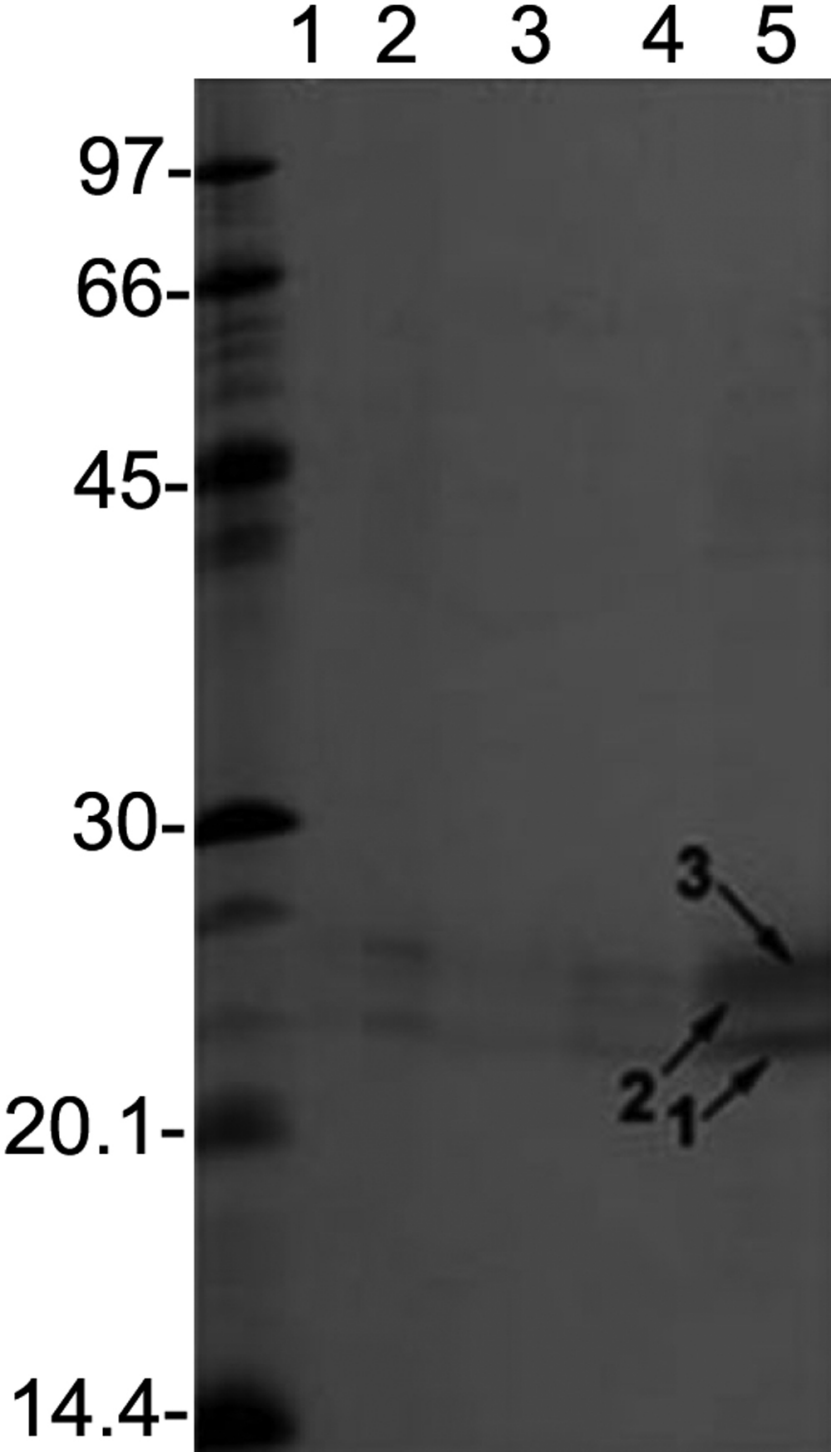
SDS–PAGE analysis of five proteinase preparations purified from five separate α-crystallin fractions. The purification was performed by the three-step procedure described in Methods. Lanes 1-5 show five different purified proteinase preparations from five separate α-crystallin fractions. Note that all five preparations reproducibly showed three bands that are identified as 1, 2, and 3.

SDS–PAGE analysis of the purified proteinase preparation showed three protein bands of 22-25 kDa (Figure 4). The five such purified preparations showed identical protein bands. The ES-MS/MS analysis identified band 1 as βA3-crystallin (contained residue #33–45 [ITIYDQENFQGKR], #35–45 [IYDQENFQGKR], #36–45 [YDQENFQGKR], #126–137 [MTIFEKENFIGR], #128–137 [IFEKENFIGR], and #197–211 [EWGSHAQTSQIQSIR]). Band 2 was identified as a mixture of βA3-, βB1-, and βB2-crystallins as it showed peptide sequences of βA3 (residue # 33–44 [ITIYDQENFQGK] and #96-109 [WDAWSGSNAYHIER]), βB1 (residue #60–71 [LVVFELENFQGR], residue #123–131 [WNTWSSSYR], #150–159 [ISLFEGANFK], #187–201 [VSSGTWVGYQYPGYR], and #202–214 [GYQYLLEPGDFR]), and βB2 (residue #110–120 [LYENPNFTGKK], #129–139 [PSFHAHGYQEK], and #145–159 [VQSGTWVGYQYPGYR]). Band 3 was identified as βB1-crystallin (contained residue #60–71 [LVVFELENFQGR], #150–159 [ISLFEGANFK], #187–201 [VSSGTWVGYQYPGYR], #202–214 [GYQYLLEPGDFR], and #233–251 [DKQWHLEGSFPVLATEPPK]).

**Figure 5:**
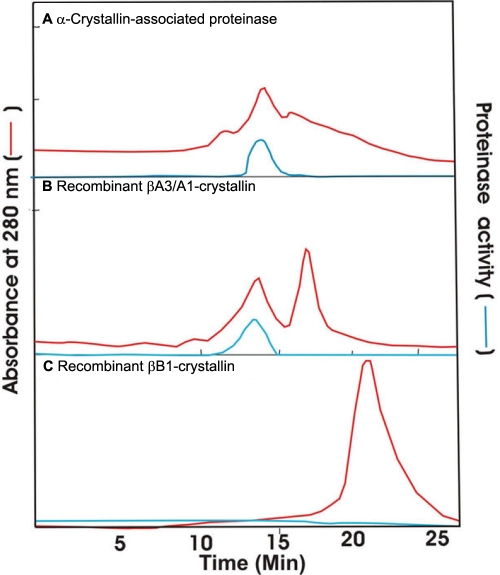
Comparison of elution profiles at 280 nm during size-exclusion HPLC  of purified proteinase from the α-crystallin fraction, recombinant βA3/A1-crystallin, and recombinant βB1-crystallin. The recombinant proteins were treated with 1% sodium deoxycholate (w/v) before their chromatography (TSK G3000 PW_XL_). The proteinase activity was determined by the incubation of column fractions with BAPNA at 37 °C for 10 h.

To determine whether the activity was associated with βA3-crystallin but not with βB1- or βB2-crystallin, the recombinant βA3- and βB1-crystallins along with purified α-crystallin proteinase were examined by HPLC using a size-exclusion TSK G-3000 PW_XL_ column. The recombinant βA3/A1-crystallin was purified as previously described [31]. The recombinant protein preparations before and after deoxycholate treatment were HPLC separated using a TSK G-3000 PW_XL_-column, and the column fractions were examined for proteinase activity. The recombinant βA3-crystallin exhibited proteinase activity, which co-eluted with proteinase purified from the α-crystallin fraction (Figure 5). No such activity was found with βB1- and βB2-crystallins (the βB2-crystallin result is not shown).

### Structural changes in βA3-crystallin following proteinase activation upon sodium deoxycholate treatment

**Figure 6:**
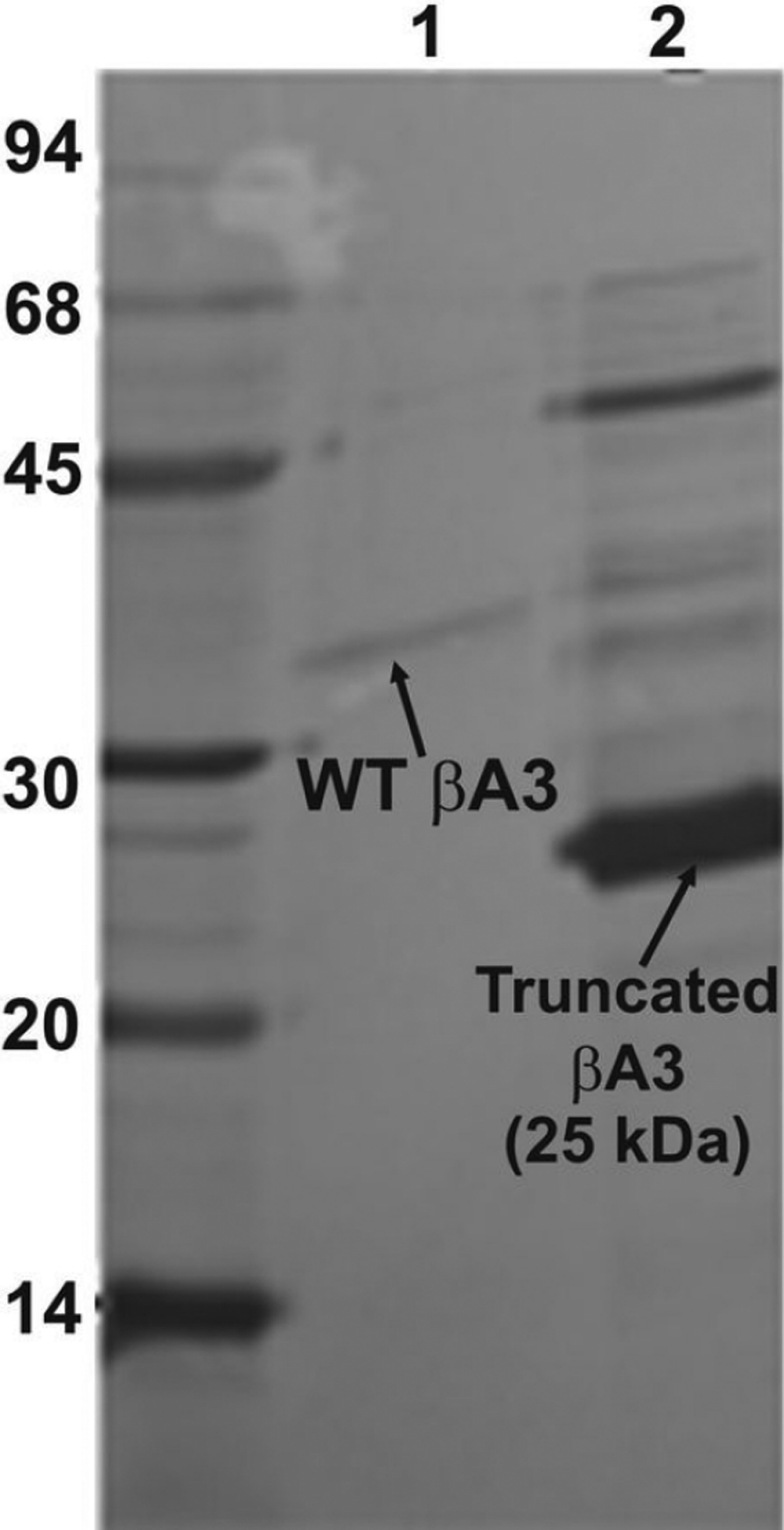
SDS–PAGE analysis of recombinant WT βA3-crystallin and after its treatment with sodium deoxycholate and size-exclusion HPLC. Lane 1 shows WT βA3-crystallin (untreated), and lane 2 shows WT βA3-crystallin with proteinase activity (after deoxycholate treatment) and size-exclusion chromatography.

**Figure 7:**
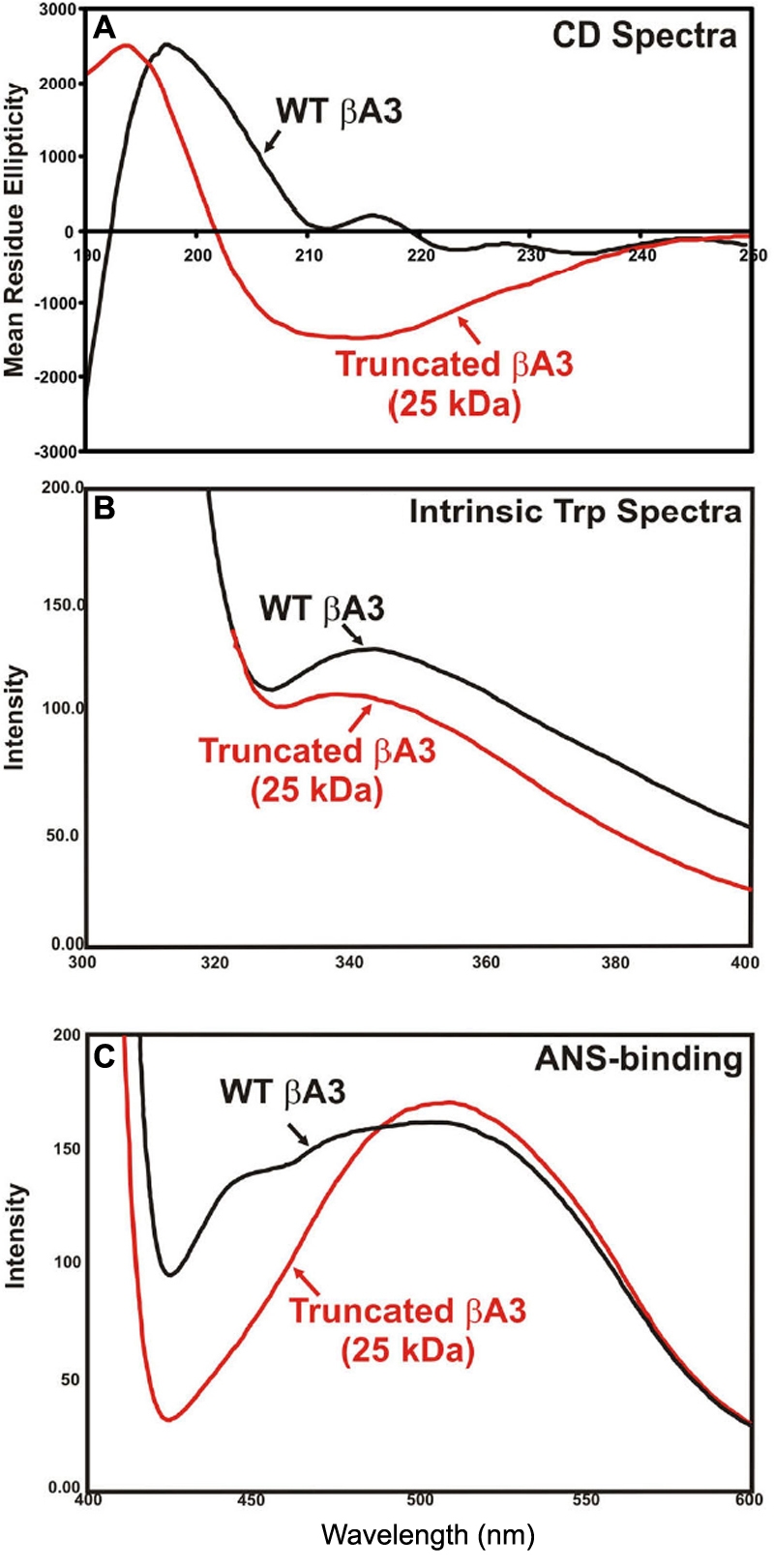
Structural changes in sodium deoxycholate-treated, 25 kDa βA3-crystallin species with proteinase activity compared to untreated, 32 kDa WT βA3-crystallin without enzyme activity. These preparations were obtained as described in Figure 6. **A**: The far UV-CD spectra show that the truncated 25 kDa βA3-species (red line) contains a greater level of alpha helical content than the WT-βA3 (black line). **B**: The intrinsic Trp fluorescence spectra show a red shift in the truncated 25 kDa βA3-species (red line) as compared to the WT-βA3 (black line). **C**: The fluorescence spectra after ANS-binding show a blue shift in the truncated 25 kDa βA3-species (red line) as compared to the WT-βA3 (black line).

Upon activation of the His-tagged WT βA3-crystallin (M_r_ ~32 kDa, Figure 6, lane 1, NH_2_-terminal sequence: MRGSH [the sequence represented His-tag]) with deoxycholate and size-exclusion HPLC, the proteinase activity-containing fraction showed an NH_2_-terminally cleaved major, 25 kDa βA3 species (Figure 6, lane 2; NH_2_-terminal sequence: MAQTN) and several aggregated species. The partial NH_2_-terminal sequencing showed that the 25 kDa species was generated upon cleavage at the K_17_-M_18_ bond in the NH_2_-terminal extension arm of WT βA3-crystallin. This cleavage site was different than the cleavage site (N_22_-P_23_) that we have reported earlier [20] during the proteinase activation. This difference could be due to the His-tag in the WT βA3-crystallin. The truncated 25 kDa species predominantly contained an α-helical structure compared to the β-sheet structure in the untruncated 32 kDa WT βA3-crystallin (Figure 7A; WT βA3: β-sheet 51% and α-helix 34%; truncated 25 kDa βA3 with proteinase activity: β-sheet 11% and α-helix 86%). Similarly, compared to the 32 kDa WT βA3-crystallin, the truncated 25 kDa βA3-crystallin species exhibited a red shift in its intrinsic Trp fluorescence spectrum (Figure 7B) and a blue shift during binding to a hydrophobic probe ANS (8-anilino-1 naphthalene sulfate; Figure 7C).

### A single Arg-bond hydrolyzing proteinase exists in different fractions of human lenses

The four experiments as described below suggested that the same Arg-bond hydrolyzing proteinase exists in the WS protein, α-crystallin, β_H_-crystallin, and membrane fractions of human lenses. Six pooled lenses of 30 to 40-year-old donors were used in these experiments.

**Figure 8:**
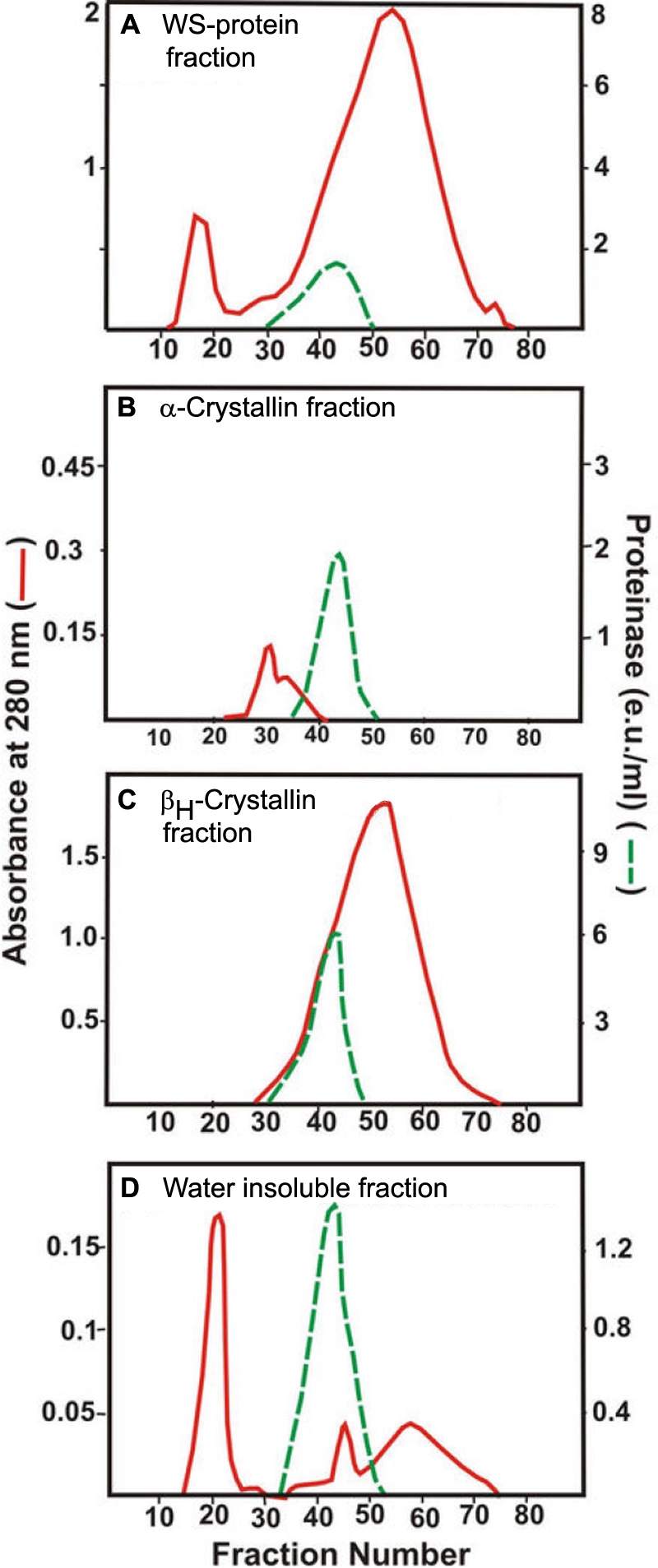
Proteinase activity in four fractions (WS proteins, α-crystallin, β_H_-crystallin, and membrane fractions) following treatment with sodium deoxycholate and size-exclusion Agarose A1.5 m chromatography. WS proteins (**A**),   α-crystallin fraction (**B**), β_H_-crystallin fraction (**C**), and membrane fraction (**D**). Each fraction was first isolated from a homogenate of six pooled lenses of donors 30–40 years old by Agarose A1.5 m chromatography. Next, each fraction was treated with the detergent and fractionated by the size-exclusion chromatography, and the column fractions were examined for their absorbance at 280 nm (red line) and proteinase activity (green line) with BAPNA as a substrate. Note that the proteinase activity eluted in the identical fractions during chromatography from each of the four fractions.

Following size-exclusion Agarose A1.5 m chromatograph of either the WS protein, α-crystallin, or β_H_-crystallin fractions, very little proteinase activity was observed in the column fractions with either the colorimetric or fluorometric substrates. However, after treatment with sodium deoxycholate and fractionation by a size-exclusion Agarose A1.5 m column, each fraction showed enzyme activity. The enzyme from each of the four fractions eluted in fractions 35-50 (in the β_H_-crystallin region), suggesting their identical molecular weights (Figure 8A-D).

**Figure 9:**
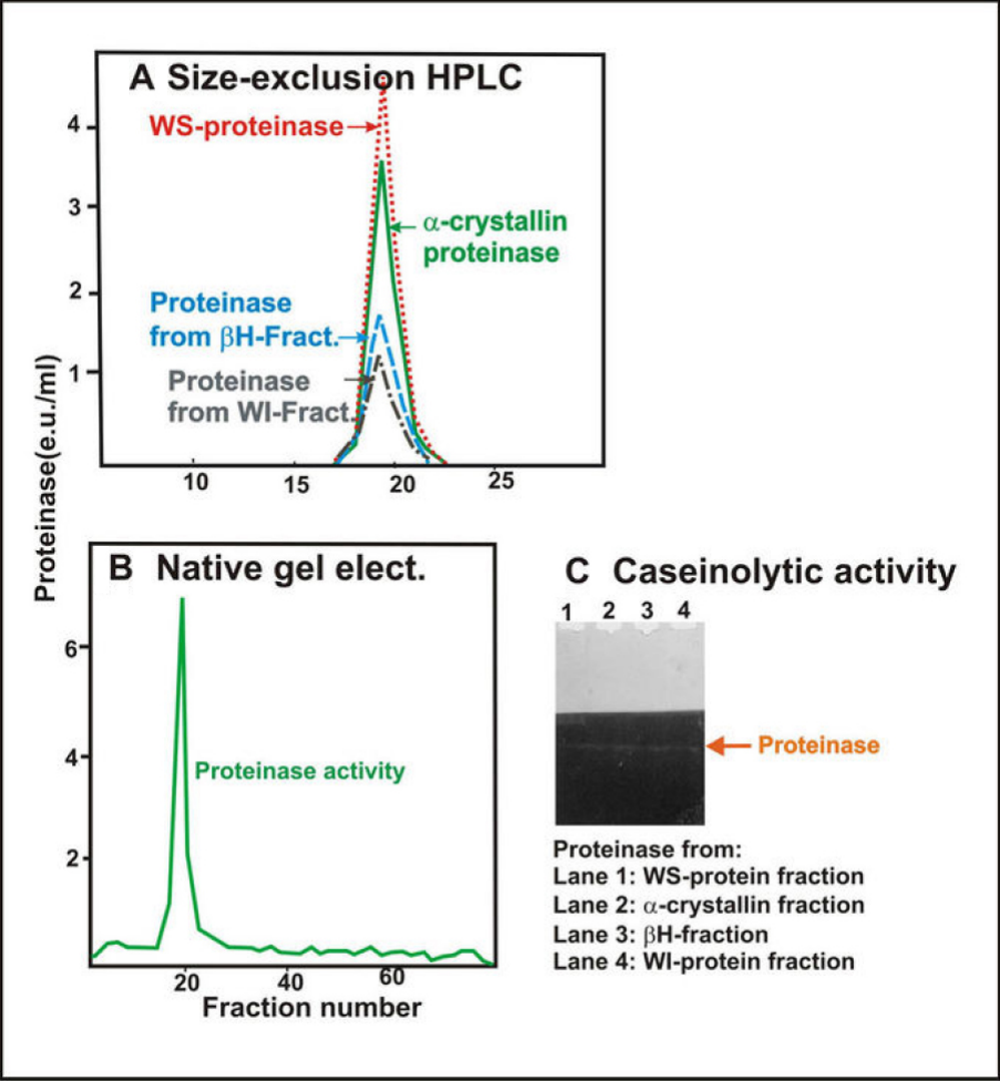
Comparison of properties of Arg-bond hydrolyzing proteinase isolated from the WS protein fraction, the α-crystallin fraction, the β_H_-crystallin fraction, and the membrane fraction of human lenses. **A**: Size-exclusion HPLC analysis is shown of the Arg-bond hydrolyzing proteinase purified from the WS protein fraction (red dotted line), the α-crystallin fraction (green dotted line), the β_H_-crystallin fraction (blue dotted line), and the membrane fraction (black dotted line). **B**: Nondenaturing gel electrophoresis is shown of the combined proteinase preparations from the WS protein fraction, α-crystallin fraction, β_H_-crystallin fraction, and membrane fraction. Note that a single proteinase peak was observed on combining proteinases isolated from all four fractions. **C**: Caseinolytic activity of the four proteinases isolated from WS-protein fraction (lane 1), α-crystallin fraction (lane 2), β_H_-crystallin fraction (lane 3), and membrane fraction (lane 4). Note that a co-migration caseinolytic activity of the four proteinases on a non-denaturation gel was observed.

 To further determine whether the enzyme from the above four fractions had an identical molecular weight, fractions 35-50 (Figure 8A-D), recovered after Agarose chromatography from each of the four fractions, were pooled separately and analyzed by size-exclusion HPLC using a TSK-G-4000 PW_XL_ column. Again, the proteinase activity from the four fractions co-eluted (Figure 9A), suggesting their identical molecular weights.

It was determined by mixing and non-denaturing gel electrophoresis that the four proteinase preparations, which were isolated from the above four fractions, co-eluted. Aliquots from proteinase fractions 35-50 of the four lens fractions (Figure 8A-D) were combined. Upon analysis by a preparative nondenaturing gel electrophoresis using a 10% polyacrylamide gel, a single proteinase activity peak was observed (Figure 9B).

To exclude the possibility that one or more of the proteinase may have degraded during the above nondenaturing gel electrophoresis, the caseinolytic activity of individual proteinase from the four fractions following nondenaturing gel electrophoresis was determined. As shown in Figure 9C, the proteinase from all four fractions showed co-migrating caseinolytic activity bands. Together, the above data suggest that the same proteinase exists in all four fractions.

### Characterization of properties of the α-crystallin-associated proteinase

#### Effect of proteinase inhibitors on α-crystallin-associated proteinase

The pI of the α-crystallin proteinase was found to be between 4.5 and 5.0. When incubating the purified proteinase with metalloproteinase, cysteine proteinase, and serine proteinase inhibitors, only the serine-proteinase inhibitors exhibited an inhibition of the enzyme, suggesting its serine-type nature.

#### Proteolysis of bovine recombinant γ-crystallins

**Figure 10:**
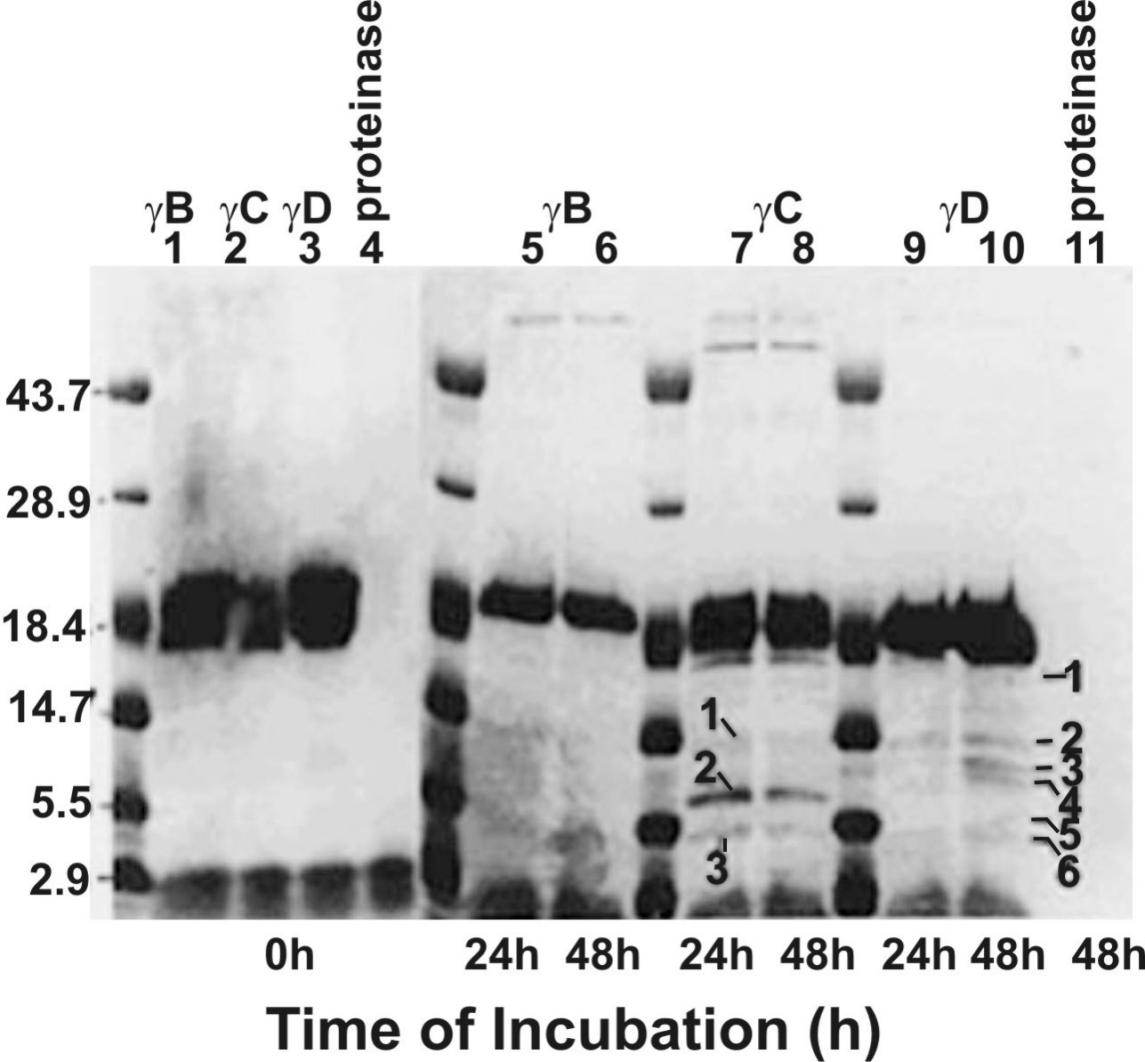
Time-dependent proteolysis of bovine recombinant γB-, γC-, and γD-crystallins by the human lens membrane proteinase. The incubation periods are shown at the bottom of the gel, and the reaction constituents are shown at the top of the gel. The proteolyzed fragments are identified with numbers, and only the fragments from γD-crystallin were used for partial NH_2_-terminal sequence analysis.

Proteolysis of the bovine recombinant γB-, γC-, and γD-crystallins was examined by the proteinase (isolated from the lens membrane fraction). As stated above in Methods, individual crystallins were incubated at the ratio of 1:40 (proteinase:crystallin) for up to 48 h at 37 °C. After withdrawing the aliquots at 0 h, 24 h, and 48 h during proteolysis and analysis by SDS–PAGE, the γB-crystallin exhibited resistance to proteolysis by the enzyme, but both γC- and γD-crystallins were proteolyzed producing three and six major fragments, respectively (Figure 10, lanes 7 and 10).The partial NH_2_-terminal sequence analysis of γD-crystallin fragments showed that the following peptide bonds were cleaved: M_1_-G_2_ in fragment #1 (~19-kDa), Q_54_-Y_55_ in fragment #2 (~14-kDa), M_70_-G_71_ in fragment #3 (~12-kDa), and Q_103_-M_104_ in fragment #4 (~10 kDa). The cleavage sites in fragments #5 and #6 could not be determined because of the presence of multiple polypeptides. In summary, the major cleavage sites in γD-crystallin by the proteinase were at M_1_-G_2_, Q_54_-Y_55_, M_70_-G_71_, and Q_103_-M_104_.

## Discussion

There were four important findings in this study: (1) The βA3-crystallin proteinase existed in an inactive state in the α-crystallin fraction, and the enzyme was activated following detergent treatment. (2) The recombinant βA3-crystallin when activated with sodium deoxycholate and size-exclusion HPLC exhibited proteinase activity, which was accompanied by cleavage at K_17_-M_18_ bond in the NH_2_-terminal extension arm. The appearance of proteinase activity was accompanied by secondary and tertiary structural changes. (3) The enzyme was present in three separate fractions (α-crystallin, β_H_-crystallin, and membrane fractions) of human lenses. (4) The proteinase exhibited in vitro proteolysis of human αA- and αB-crystallins and bovine γC- and γD-crystallins. The first finding suggests the potential role of α-crystallin as a proteinase inhibitor as evident from previous studies [23,24,26]. The second finding suggests that the activation of the proteinase activity in the recombinant βA3-crystallin was accompanied by cleavage in the NH_2_-terminal arm and secondary structural changes, which support our previous finding that truncation in the NH_2_-terminal arm was needed for the activation of proteinase activity in the human lens βA3-crystallin [20]. The third finding of the presence of the same enzyme in the α-crystallin and membrane fractions in addition to the β_H_-crystallin fraction suggests an age-related aggregation of βA3-crystallin with α-crystallin and later the localization of the aggregate with the membrane. The fourth finding suggests that the enzyme, although remaining in an inactive state in the α-crystallin fraction, is capable of proteolyzing αA-, αB-, and γ-crystallins. These findings are significant in view of our present limited knowledge about in vivo physiologic and biochemical relevance of βA3-crystallin proteinase activity.

Our studies have shown that αA- and αB-crystallins form aggregates with βA3-crystallin, which occurs due to hydrophobic interaction (unpublished results). We hypothesize that with deoxycholate treatment, the enzyme dissociated from α-crystallin because of its greater solubility in the detergent compared to α-crystallin. Once released from the α-crystallin fraction upon detergent treatment, the enzyme elutes as a lower molecular weight species (in the β_H_-crystallin region) compared to the α-crystallin. A previous report has shown that sodium deoxycholate treatment disrupted the native oligomeric structures of both αA- and αB-crystallins and resulted in their tetrameric state without significant changes in their secondary structures [37]. However, we did not observe the tetrameric state of α-crystallin. Instead, the crystallin eluted in the void volume during size-exclusion Agarose A1.5 m chromatography. This could be due to the removal of the detergent from α-crystallin following chromatography that might have led to the reorganization of the α-crystallin oligomer back to the original state. Indeed, the excess of detergent was recovered as a separate peak after the elution of the proteinase during Agarose A1.5 m chromatography. Previous reports have suggested potential binding of proteins via hydrophobic interactions with α-crystallin. The labeling of an intrinsic membrane protein with the probe, trifluoromethyl-3-(~z-[' I] iodophenyl) diazirine, showed that α-crystallin contains hydrophobic regions on its surface, which might enable it to make contact with the lipid bilayer [38]. The interactions between α-crystallin and the membrane is not membrane lipid-specific but rather α-crystallin aggregate-specific [39], and the binding of αA- and αB-crystallins to the lens membrane is via hydrophobic interactions [40]. Carver et al. [41] have suggested that α-crystallin acts as an anionic surfactant during interaction with proteins to prevent precipitation of the partially unfolded form of proteins. Furthermore, the binding of α-crystallin to form a complex with a proteolytic enzyme has also been shown, i.e., a complex formation with prochymosin during refolding but not with active chymosin [42]. Prochymosin is a zymogen of chymosin, which is a milk-clotting enzyme with low proteolytic activity. Previously, our results showed that deoxycholate treatment leads to a redistribution of different species of the human lens β-crystallin as represented by the generation of new aggregates, and these species were originally not present in detergent-untreated β-crystallins [20]. Taken together, the association of proteinase with α-crystallin seems to be through hydrophobic interactions, which resulted in the release of the proteinase when disrupted by a detergent. Although proteolysis of both αA- and αB-crystallins was seen through in vitro incubation, whether the enzyme activity was acquired before or after dissociation from α-crystallin is presently unclear.

The proteinase was purified about 500 fold with a 1,094 fold increase in specific activity following the three-step purification method. SDS–PAGE analysis on the highly purified proteinase preparation showed three protein bands, which were identified as βB1-, βB2-, and βA3-crystallins by the ES-MS/MS mass spectrometric method. Among these crystallins, only recombinant βA3-crystallin showed proteinase activity. The recombinant human βA3-crystallin used in this study was purified to almost homogeneity by a Ni^2+^-affinity column and showed only a single band of 32 kDa during SDS–PAGE and a single spot on a two-dimensional gel electrophoretic analysis (results not shown). Detergent treatment and the activation of the enzyme activity in the crystallin resulted not only in its degradation but also the presence of several yet to be identified species. These species could be degradation products of the crystallin and their multimers.

It was surprising that the purified proteinase showed a complex of βB1-, βB2-, and βA3-crystallins, and the complex of the three crystallins remained together during the three purification steps. Our attempts to dissociate the three crystallins from the complex by an ion-exchange column chromatographic method were unsuccessful. However, such complexes with αA- and αB-crystallins in the HMW proteins of both young and old human lenses were shown in our recent study [43].

As stated above, α-crystallin showed inhibition of both trypsin and elastase during in vitro experiments [24,26]. Our recent studies about the interaction of human αA- or αB-crystallins with βA3-crystallin through the two-hybrid system and in vivo FRET (fluorescence resonance energy transfer) have also suggested interactions among the crystallins (unpublished results). Therefore, we speculate that the inhibition of the enzyme by α-crystallin might be an in vivo mechanism to prevent unwanted proteolysis of αA- and αB-crystallins and to preserve their chaperone activity. Such speculation is strengthened by the fact that the same proteinase was present in an inactive state in α-crystallin, β_H_-crystallin, and membrane fractions of human lenses. This could be due to age-related distributional changes in a complex with α-crystallin and βA3-crystallin. This is also consistent with our results showing that βA3-crystallin exists in both urea soluble and urea insoluble fractions of the WI protein fraction of human lenses [17,18]. Although the membrane fraction was prepared by the extensive urea washing method as described in the literature [6], a need for activation of the proteinase by a detergent from this fraction suggests the existence of an inactive enzyme.

Human βA3/A1-crystallin undergoes several posttranslational modifications that include S-methylation, glutathiolation, and truncation [44]. A loss 4 and 18 NH_2_-terminal residues in βA3/A1-crystallin at an early stage of life (i.e., three years of age) has been shown [16,19]. This truncation of the NH_2_-terminal extension at a very early developmental stage does not significantly alter the structural properties. Further, three of the five NH_2_-terminal truncations in the crystallin were found to be adjacent to a proline residue (i.e., N_4_-P_5_, P_7_-G_8_, and G_10_-P_11_) [45]. A comparison of nuclear and cortical βA3/A1-crystallins from 11-year-old and 19-year-old human lenses showed more truncation among nuclear than cortical βA3/A1-crystallins in both ages [44]. Our results also showed that the majority of age-related degradation in human βA3/A1-crystallins occurred at the NH_2_-terminal region with a major cleavage site at the E_39_-N_40_ bond [15]. Recently, we identified several truncated species of βA3/A1- and βB1-crystallins in human cataractous lenses that were absent in normal lenses [17,18]. Because our recent study [31] showed that the NH_2_-terminal domain is relatively more stable than the COOH-terminal domain in human βA3-crystallin, the truncation of the NH_2_-terminal regions might impair its stability to form complexes with other β-crystallins and might lead to aggregation with α-crystallin. As stated above, the Arg-bond hydrolyzing activity is associated with a truncated βA3/A1-crystallin [20], and therefore, the regulation of the enzyme activity in vivo might involve the prevention of truncation of βA3/A1-crystallin and the inhibition of the enzyme activity of the truncated crystallin.

Several important questions emerge from this study. The activation mechanism of the crystallin proteinase and its association with different crystallins including the lens membrane remain to be studied. Further, an understanding of the molecular mechanism of the proteinase inhibition by α-crystallin in relation to the chaperone function of the latter is needed. In future studies, we will utilize α-crystallin-associated proteinase as the model system to answer these questions.
